# Spinal anesthesia and hypotensive events in hip fracture surgical repair in elderly patients: a meta-analysis

**DOI:** 10.1186/s44158-022-00047-6

**Published:** 2022-05-08

**Authors:** Antonio Messina, Luigi La Via, Angelo Milani, Marzia Savi, Lorenzo Calabrò, Filippo Sanfilippo, Katerina Negri, Gianluca Castellani, Gianmaria Cammarota, Chiara Robba, Emanuela Morenghi, Marinella Astuto, Maurizio Cecconi

**Affiliations:** 1 Humanitas Clinical and Research Center – IRCCS, Milano, Italy; 2 Department of Biomedical Sciences, Humanitas University, Pieve Emanuele, MI Italy; 3 Department of Anaesthesia and Intensive Care, A.O.U. “Policlinico-San Marco”, Via Santa Sofia 78, 95123 Catania, Italy; 4 Department of Anesthesia and Intensive Care Medicine, Maggiore della Carità University Hospital, Novara, Italy; 5 Anesthesia and Intensive Care, San Martino Policlinico Hospital, IRCCS for Oncology and Neuroscience, Genoa, Italy; 6 School of Anaesthesia and Intensive Care, University Hospital “G. Rodolico”, University of Catania, Catania, Italy

**Keywords:** Hypotension, Spinal anesthesia, Hip fracture

## Abstract

**Background:**

Spinal anesthesia (SA) is widely used for anesthetic management of patients undergoing hip surgery, and hypotension is the most common cardiovascular side effect of SA. This paper aims to assess the lowest effective dose of SA that reduces the occurrence of intraoperative hypotension in elderly patients scheduled for major lower limb orthopedic surgery.

**Methods:**

We conducted a systematic review of randomized controlled trials (RCTs) performed in elderly patients scheduled for surgical hip repair and a meta-analysis with meta-regression on the occurrence of hypotensive episodes at different effective doses of anesthetics. We searched PUBMED®, EMBASE®, and the Cochrane Controlled Clinical trials registered.

**Results:**

Our search retrieved 2085 titles, and after screening, 6 were finally included in both the qualitative and quantitative analysis, including 344 patients [15% (10–28) males], with a median (25th to 75th interquartile) age of 82 (80–85). The risk of bias assessment reported “low risk” for 5 (83.3%) and “some concerns” for 1 (16.7%) of the included RCTs.

The low dose of SA of [mean 6.5 mg (1.9)] anesthetic was associated with a lower incidence of hypotension [OR = 0.09 (95%CI 0.04–0.21); *p* = 0.04; *I*^2^ = 56.9%], as compared to the high-dose of anesthetic [mean 10.5 mg (2.4)].

**Conclusions:**

In the included studies of this meta-analysis, a mean dose of 6.5 mg of SA was effective in producing intraoperative comfort and motor block and associated with a lower incidence of hypotension as compared to a mean dose of 10.5 mg.

**Trial registration:**

CRD42020193627

**Supplementary Information:**

The online version contains supplementary material available at 10.1186/s44158-022-00047-6.

## Introduction

Hip fracture is a significant cause of morbidity and mortality and a common reason for older and fragile people to require emergency surgery and hospital admission [1]. Spinal anesthesia (SA) is widely used for anesthetic management of patients undergoing lower limbs orthopedic procedures, such as hip fracture repair [2, 3]. However, anesthesia for hip fracture repair is remarkably variable, making the most effective choice between SA and general anesthesia still debated [4, 5], despite an increasing worldwide trend in the use of SA during the last two decades [1, 6]. SA seems to reduce specific postoperative outcomes (such as hospital length of stay and cardiopulmonary complications) after hip fracture surgery [7, 8], especially in sicker and older patients [9], except for long-term morbidity and mortality [1, 10]. In fact, recent consensus recommendations based on a large systematic review and meta-analysis strongly suggest using neuraxial over general anesthesia for primary unilateral total hip arthroplasty [11].

There are several different complications described in the literature following SA. Those related to local tissues or spinal cord damage [12–15] are very rare, whereas those associated with the systemic effect of the drugs injected are more common [12, 15, 16]. Among the latter, SA cardiovascular side effects predominate, with hypotension being the most frequent, occurring in up to 33% of cases [16, 17]. SA affects sympathetic chain activity, leading to a reduction in vasomotor tone, in turn in preload (due to venodilation, resulting in decreased venous return), afterload (reduced systemic vascular resistances) [15, 18], and finally, in cardiac output, especially in the elderly population [19]. As a matter of fact, autonomic nervous system function plays a key role in the development of hemodynamic instability and intraoperative hypotension [20], which is a well-established source of postoperative complications [21, 22]. Hemodynamic stability should be considered as a primary intraoperative target, since several findings suggest avoiding systemic pressure drops [i.e., the mean arterial pressure above 65 mmHg], even for a few minutes [21, 22], and the overall intraoperative trends in the mean arterial blood pressure (i.e., the sum of consecutive jumps or drops across a surgery) is independently associated with -day mortality [23].

Accordingly, assessing the optimal dose that allows for surgery and guarantees patient comfort without compromising the cardiovascular system is demanding [22]. Hypotension is primarily related to the overall dose injected [15]; however, several other variables, including the volume, the type of the anesthetics injected, different adjuvant agents, and pre- and intraoperative factors, may impact the hemodynamic effect of the SA [15, 24].

Therefore, we conducted a systematic review of randomized-controlled trials (RCT) and performed a meta-analysis with meta-regression to assess the lowest effective dose of SA (i.e., determining a successful sensory and motor block) that reduces intraoperative hypotension in elderly patients scheduled for major lower limb orthopedic surgery. Secondarily, we conducted a meta-regression to assess the impact of predefined SA and patient variables on the occurrence of hypotensive events.

## Material and methods

We adhered to the *Preferred Reporting Items for Systematic Reviews and Meta-Analysis - Protocols* (PRISMA-P) guidelines (Supplemental Table 1 in the Online Supplemental Materials). This study’s protocol was registered with the *International Prospective Register of Systematic Reviews* (PROSPERO) in July 2020 (CRD42020193627—data screening start: 30/08/2020; data extraction start: 30/09/2020).

### Data sources and search strategy

A senior author (A.M.) performed the article search through EMBASE (including EMBASE® and MEDLINE®) and the Cochrane Database of Systematic Reviews without crowdsourcing, using the following keywords and their related MeSh terms: “hypotension” AND “spinal anesthesia,” restricting the search to studies performed in human adults. The complete systematic review search strategy is reported in Supplemental Table 2 in the Online Supplemental Materials. We included RCTs reporting the use of different doses of the same SA anesthetic agent for open surgical repair of hip fracture, published in the English language, in indexed scientific journals, from 1 January 1990 up to 31 January 2020. Moreover, to be included, the studies had to state no difference in terms of SA efficacy (i.e., successful sensory and motor blocks associated with no need for adjunctive deep sedation and/ or general anesthesia to guarantee intraoperative patient comfort) between the groups.

Moreover, we excluded RCTs using combined techniques (i.e., spinal-epidural) or more than a single spinal anesthetic agent and all studies using spinal or intravenous vasoactive agents pre-SA or SA sympathomimetic adjuvants, administered with the stated purpose of reducing post-SA hypotension.

Considering the variability in the definition of hypotension in the intraoperative hypotension in the literature, we restricted the inclusion to those studies reporting an episode of hypotension as systolic blood pressure (SBP) < 90 mmHg or as a reduction of at least 20% from baseline values which are of two of most frequently used definitions of intraoperative hypotension in the literature [23, 25, 26].

Inclusion criteria for clinical studies were pre-specified according to the PICOS approach:

- P: patients scheduled for surgical repair of hip fracture
- I: receiving SA for the surgery
- C: comparison between groups regarding the incidence of post-SA
- O: data provided according to SA technique, baseline patients’ characteristics
- S: RCTs reporting the use of different doses of the same SA anesthetic

### Data abstraction and quality assessment

Two examiners (An.Mi. and M.F.) independently evaluated the titles and abstracts. The articles were then subdivided into three subgroups: “included” and “excluded” (if the two examiners agreed with the selection) or “uncertain” (in case of disagreement). In the case of “uncertain” classification, discrepancies were resolved by further examination performed by an expert author (A.M. or M.C.). We used a standardized electronic spreadsheet (Microsoft Excel, V 14.4.1; Microsoft, Redmond, WA) to extract the data from all included studies, recording: trial characteristics (i.e., number centers involved, the period of enrolment), patient population (i.e., demographics, type of surgery, baseline illness severity scores), description of SA technique (i.e., *the* modality of administration, an anesthetic drug, dose, volume, site of puncture), hemodynamic parameters recorded before and after SA, and the complications related to SA administration (i.e., hypotension, bradycardia) (Supplemental Table 3 in the Online Supplemental Materials). When necessary, the included studies’ corresponding authors were contacted to obtain missing data about trial demographics, methods, and outcomes.

### Outcomes

Our primary outcome was to appraise the association between the dosage of local spinal anesthetic and the occurrence of hypotensive events in the enrolled population (i.e., the rate of enrolled patients in each subgroup having at least one episode of hypotension (Supplemental Table 4 in the Online Supplemental Materials).

The secondary outcome was to evaluate whether specific pre-existing patient-related or SA characteristics could affect the primary outcome.

### Risk of bias assessment in the included studies

Two senior authors (A.M. and M.C.) assessed the internal validity of those studies included in the quantitative analysis using the Cochrane Collaboration’s Risk of Bias tool (RoB-2 version 2019) [27], which provides specific criteria for appraisal of risk according to the following domains: (1) risk of bias arising from the randomization process, (2) risk of bias due to deviations from the intended interventions, (3) risk of bias due to missing outcome data, (4) risk of bias in the measurement of the outcome, and (5) risk of bias in the selection of the reported result. The overall risk-of-bias judgment has been finally provided, according to the five domains of bias assessment as “low risk,” “some concerns,” or “high risk” [27].

### Statistical analysis

Descriptive analysis was carried out: the statistical unit of observation for all the selected variables was the single study and not the patient. Normal distribution of continuous variables was evaluated by employing the d’Agostino-Pearson test, and data expressed as mean (standard deviation, SD) or median (25–75 interquartile, IQR) appropriately.

We stratified the included studies into low dose/high dose subgroups, according to the trial definition. For those trials having more than two subgroups, we calculated the median dose of local anesthetic administrated, and then, accordingly, sorted the overall population to obtain two final subgroups (i.e., two or more subgroups receiving a dose lower or higher than the median of the single study were merged into the same subgroup).

According to the definition adopted in the study, we considered the reported rate of patients having at least one post-SA event of hypotension. Due to the imbalance between group sizes, the odds ratios (ORs) and 95% confidence intervals (CIs) were calculated using the Der Simonian-Laird method with a random-effects model. For calculated ORs, 0.5 was added to each of the four interior cells if one of the cells contained zero. Publication bias was graphically evaluated using funnel plots. Heterogeneity was measured using *Q* and *I*^2^ tests, which were considered significant when the *p*-value was < 0.1 and *I*^2^ > 50%. According to Higgins et al. [28], *I*^2^ values of 0–25%, > 25%, > 50%, and 75% represented none, low, moderate, and high heterogeneity, respectively. We then conducted a meta-regression to explore the impact of baricity of the spinal anesthetic agent used (i.e., hypobaric or hyperbaric), body mass index (BMI), preoperative American Society of Anaesthesiologists Physical Status (ASA) classification, intraoperative blood loss, pre-existing hypertension, and preoperative use of beta-blockers.

All statistical analyses were performed using Stata statistical software (version 15.0, StataCorp) and GraphPad PRISM V8 (GraphPad Software Inc., San Diego, CA, USA).

## Results

**Fig. 1:**
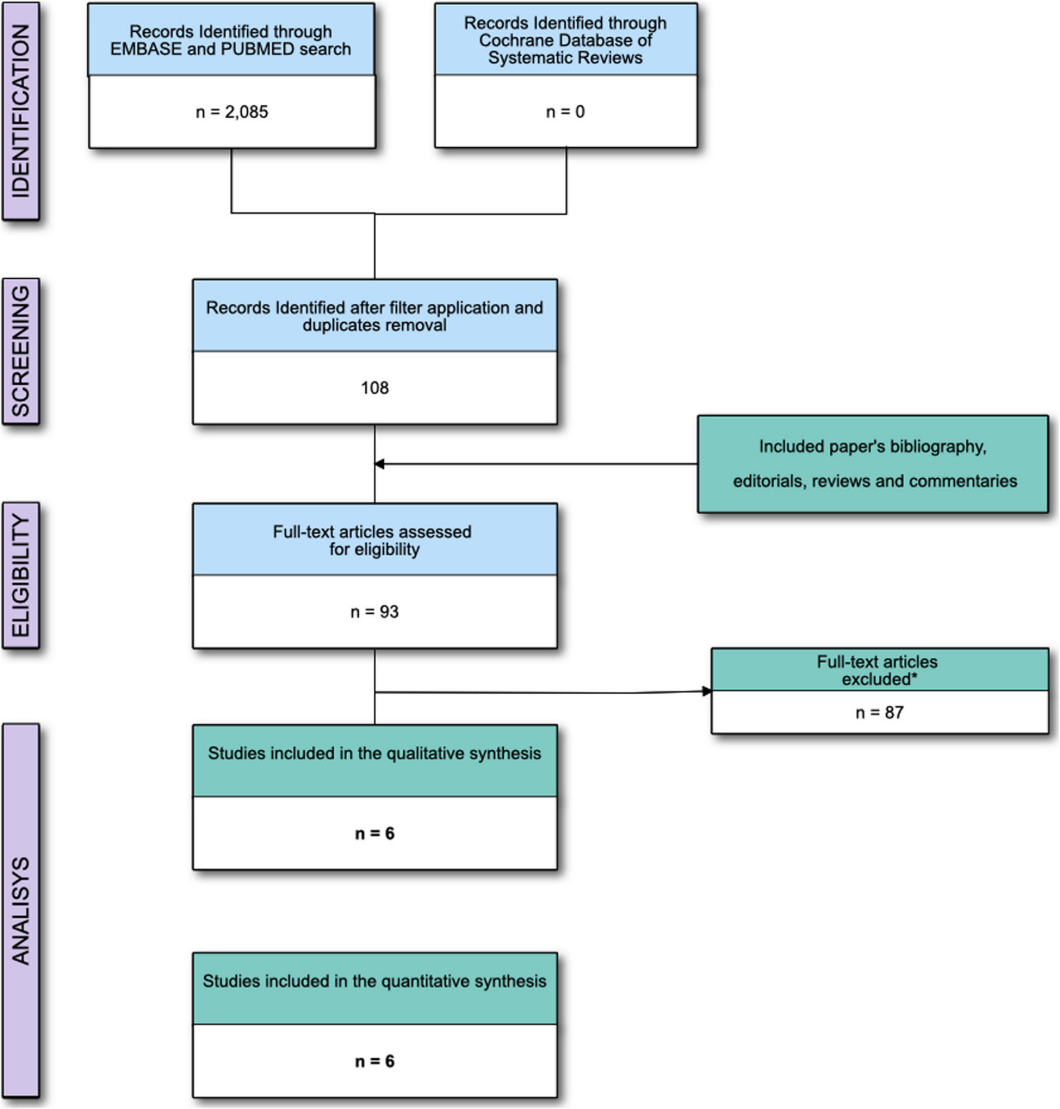
Flow of the studies. *Excluded studies are reported in Supplemental Table 4 in the Online Supplemental Materials

As shown in Fig. 1, the primary electronic search identified 2085 articles. The examiners identified 95 potentially relevant studies from the analysis of the title and abstract. Experts evaluated and solved the inclusion of 5 (5.2%) potentially relevant studies because of disagreement between the examiners. The list of the excluded studies is reported in Supplemental Table 5 in the Online Supplemental Materials.

### General characteristics of the included studies

**Fig. 2:**
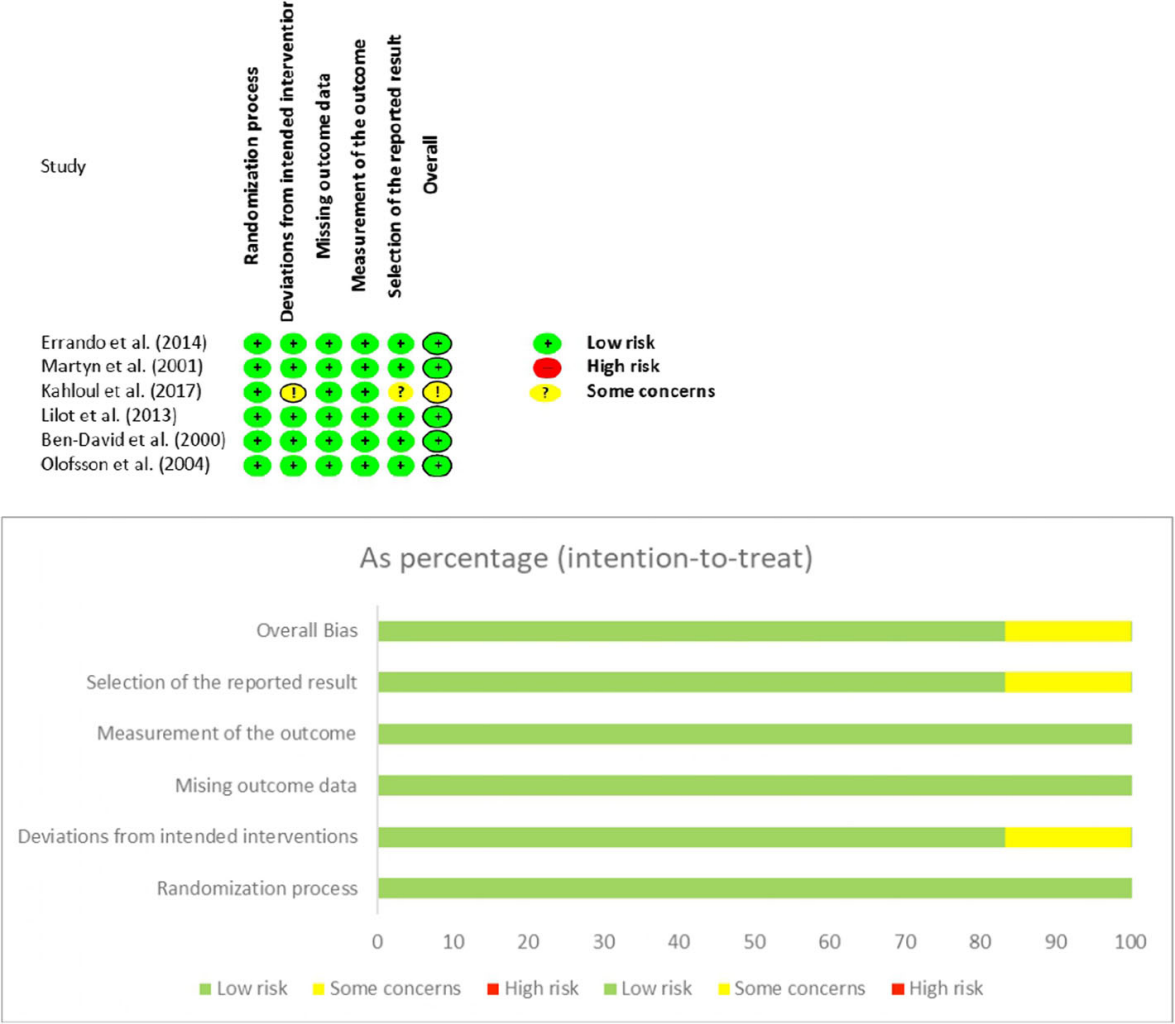
Cochrane Collaboration’s Risk of Bias assessment and summary (RoB-2 version 2019) [27]. Green circles = low bias; yellow circles = some concerns

We finally included 6 studies in both the qualitative and quantitative analysis (Table 1 and Fig. 1), including 344 patients [15% (10–28) males, 58 (35–76) patients included in each study with a median age of 82 (80–85) years, BMI of 23 (23–24) kg/m^2^]. All studies were monocentric, and only one study [33] reported the overall enrolment period (9 months). The risk of bias assessment reported “low risk” for 5 [29–32, 34] (83.3%) and “some concerns” for 1 [33] (16.7%) of the included RCTs (Fig. 2 and Supplemental Table 6). None were at a high risk of bias. Except for two studies [31, 33], the others reported a median pre-SA SBP of 155 (152–158) mmHg. All the studies, but one [33], infused a median of 373 (293–494) ml of fluid before or during SA administration. The median length of the surgery was 65 (56–78) min. Hypotensive episodes were managed in all the studies with boluses of 5–10 mg of ephedrine, except for one study adopting boluses of 0.25 mg of metaraminol [32].

**Table 1:** Characteristics of the enrolled studies

Authors	Subgroup	Patients' characteristics								Surgery characteristics	
		Number	Age	Male (%)	BMI (kg/m^**2**^)	ASA I-II (%)	ASA III-IV (%)	Hypertension (%)	Home β-blockers’ use (%)	Surgery length (min)	Blood loss (ml)
Errando et al. [29]	A	31	79 (6)	19	24*	26	74	45	NA	59 (23)	NA
	B	30	80 (8)	27	27*	40	60	63	NA	55 (12)	NA
Ben-David et al. [30]	A	10	85 (4)	10	21*	NA	NA	NA	NA	80 (60–100)	NA
	B	10	82 (5)	10	24*	NA	NA	NA	NA	70 (50–110)	NA
Olofsson et al. [31]	A	25	76 (2)	NA	NA	100	0	NA	NA	49 (8)	NA
	B	25	78 (1)	NA	NA	100	0	NA	NA	39 (5)	NA
Martyr et al. [32]	A	20	86 (8)	35	NA	20	80	NA	NA	NA	199 (112)
	B	20	82 (6)	10	NA	15	85	NA	NA	NA	194 (119)
Kahloul et al. [33]	A	54	80 (8)	NA	24 (3)	NA	NA	NA	13	59 (26)	NA
	B	54	81 (6)	NA	23 (3)	NA	NA	NA	20	57 (17)	NA
Lilot et al. [34]	A	17	86 (6)	18	21*	59	41	77	18	45 (30–55)	NA
	B	17	85 (7)	12	24*	65	35	59	29	40 (35–50)	NA
	C	15	84 (6)	33	23*	53	47	33	7	45 (35–60)	NA
	D	16	83 (4)	13	23*	69	31	63	38	48 (38–70)	NA

Demographic and intraoperative surgical characteristics of the included studies. According to data reporting in each study, age is reported as mean (standard deviation) or median [confidence intervals]. *NA*, data not available; *BMI*, body mass index—BMI*, body mass index calculated from the reported averaged values of height and weight of the enrolled patients; *ASA*, American Society of Anesthesiologists (ASA) physical status classification

### Quantitative meta-analysis and meta-regression

**Fig. 3:**
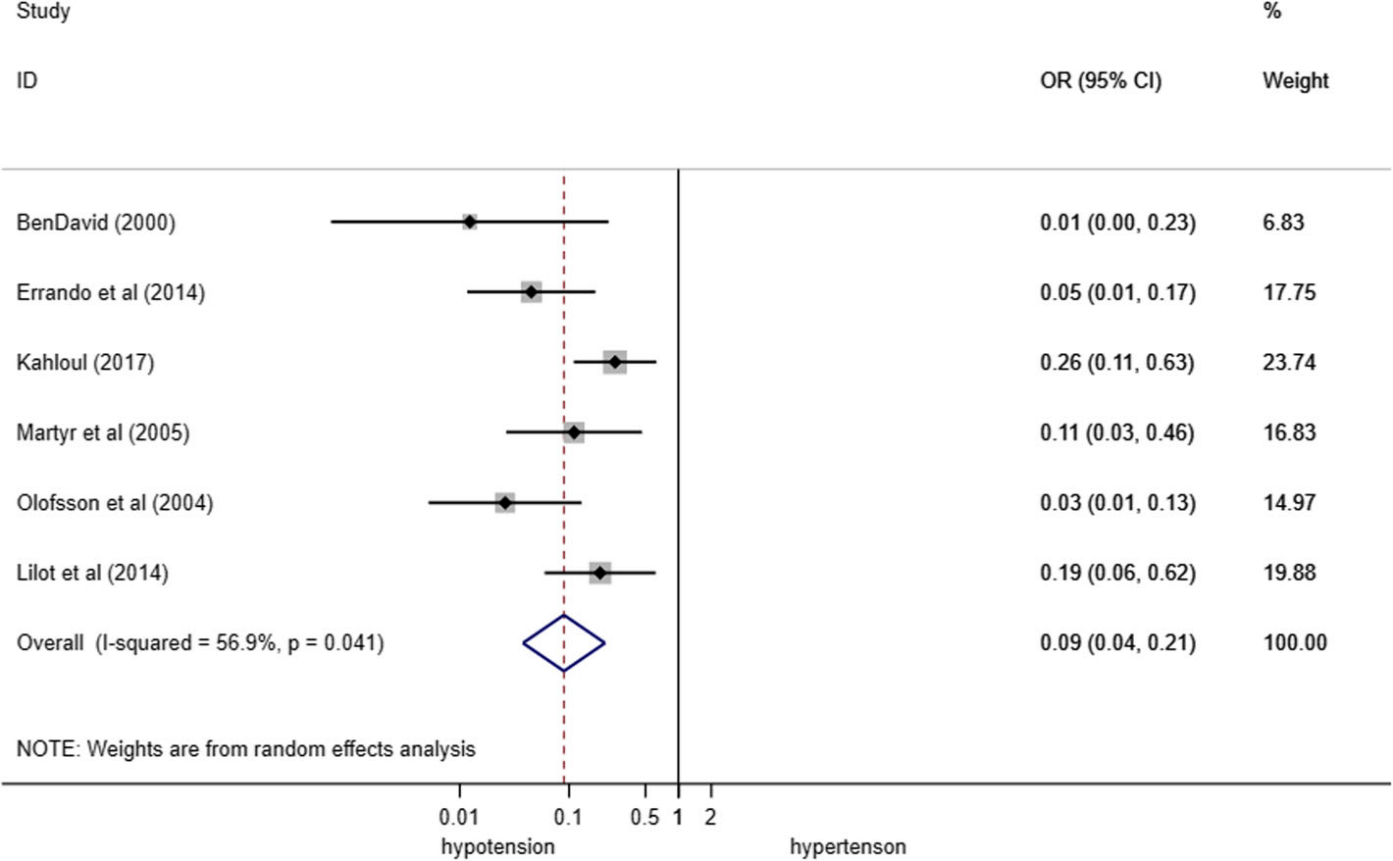
Forrest plot regarding the effect of low/high spinal anesthesia dose in the included trials included in the quantitative analysis. OR, odds ratio; CI, confidence interval

**Fig. 4:**
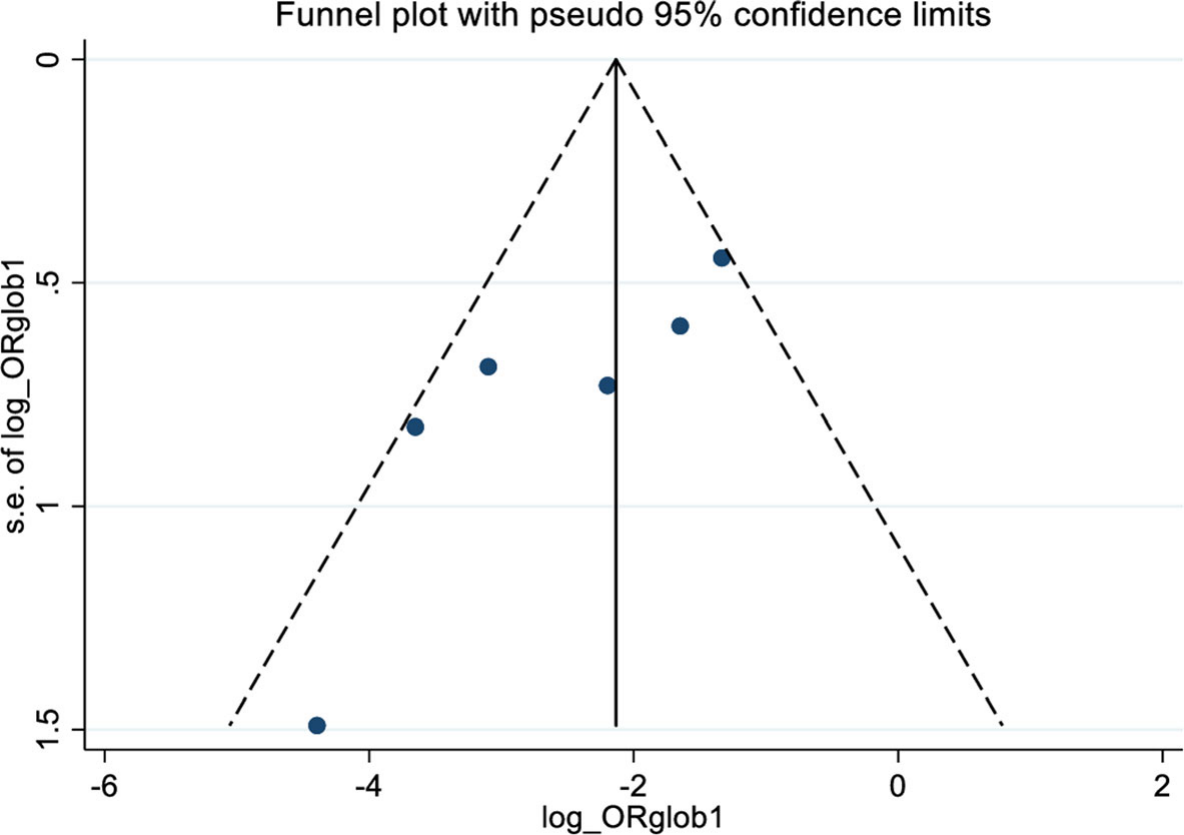
Publication bias funnel plot (with 95% confidence limits)

The overall low mean SA dose was 6.5 mg (1.9), while the high mean dose was 10.5 mg (2.4), as shown in Table 2. The low dose of anesthetics was associated with lower incidence of hypotension [OR = 0.09 (95%CI 0.04–0.21); *p* = 0.04; heterogeneity chi-squared = 11.59 (d.f. = 5); *I*^2^ = 56.9%] (Figs. 3 and 4).

**Table 2:** Characteristics of spinal anesthesia in the included studies

	Study													
	Errando et al. [29]		Ben David et al. [30]		Olofsson et al. [31]		Martyr et al. [32]		Kahloul et al. [33]		Lilot et al. [34]			
Anesthetic	Hyper Bupivacaine 0.25%	Hyper Bupivacaine 0.5%	Hyper Bupivacaine 0.5%		Hyper Bupivacaine 0.5%		Hypo Bupivacaine 0.5%		Hypo Bupivacaine 0.5%		Iso-hypo ropivacaine 0.75%			
Dose (mg)	3.75	7.5	4	10	7.5	15	9	11	5	7.5	6	8	10	12
Subgroup	L	H	L	H	L	H	L	H	L	H	L	L	H	H
Adjuvant (opiate)	N/A	N/A	Fentanyl	N/A	Sufentanil	N/A	Fentanyl	N/A	Fentanyl	Fentanyl	Sufentanil	Sufentanil	Sufentanil	Sufentanil
Adjuvant dose (mcg)	-	-	20	-	5	-	20	-	25	25	5	5	5	5
Puncture level	L2–L3 L3–L4	L2–L3 L3–L4	L3–L4	L3–L4	L2–L3	L2–L3	L3–L4	L3–L4	L3–L4 L4–L5	L3–L4 L4–L5	L3–L4 L4–L5	L3–L4 L4–L5	L3–L4 L4–L5	L3–L4 L4–L5
Needle size	25 G	25 G	22 G	22 G	27 G	27 G	22 G	22 G	-	-	25 G	25 G	25 G	25 G
Infused volume (ml)	1.5	1.5	2	4	-	-	2.2	2.2	3	3	-	-	-	-
Position during procedure	Lateral on the operating side	Lateral on the operating side	Lateral, fracture side up	Lateral, fracture side up	Sitting	Sitting	Lateral, fracture side up	Lateral, fracture side up	Lateral, fracture side up	Lateral, fracture side up	Lateral, fracture side up	Lateral, fracture side up	Lateral, fracture side up	Lateral, fracture side up
Level of sensory block (max)	T10	T10	T8	T6	T7	T6	T6–T12	T5–L3	-	-	-	-	-	-
Sensory block onset (min)	-	-	-	-	-	-	-	-	8 (4)	6 (2)	-	-	-	-
Motor block onset (min)	-	-	-	-	-	-	-	-	-	-	-	-	-	-
Duration sensory block (min)	78.6 [60–160]	125.5 [70–210]	-	-	-	-	-	-	91 (32)	113 (19)	105 [100–200]	120 [105–160]	140 [110–160]	133 [108–170]
Duration motor block (min)	-	-	-	-	-	-	-	-	-	-	115 [100–125]	120 [105–131]	150 [110–160]	123 [108–213]
Modified Bromage scale (0–3)	-	-	-	-	2	3	-	-	-	-	-	-	-	-
Type of fluid infused	RL	RL	RL	RL	RL	RL	HaS	HaS	NS	NS	HES	HES	HES	HES
Dose of infused fluid (mg/kg)	-	-	8	8	-	-	8	8	5	5	5	5	5	5
Total fluid administered (ml)	500	500	-	-	500	500	-	-	-	-	-	-	-	-
**Patients with hypotensive events (%)**	14	76	10	90	16	88	25	75	59	92	53	47	83	81

*Hyper* hyperbaric, *Hypo* hypobaric, *Iso* isobaric, *L* low-dose subgroup, *H* high-dose subgroup, *N/A* not administered, *G* gauge, *RL* Ringer Lactate, *HaS* Hartmann’s solution, *NS* normal saline, *HES* hydroxyethyl starch 6%. Bromage motor blockade score: grade I, free movement of legs and feet; grade II, just able to flex knees with free movement of feet; grade III, unable to flex knees, but with free movement of feet; and grade IV, unable to move legs or feet. The fluids infused before spinal anesthesia are reported as either a standardized bolus (i.e., 500 ml for all the enrolled patients) or a weight-based bolus (5–8 ml/hg)

The meta-regression showed that hyperbaric SA was associated with a higher rate of hypotension [OR = 1.85 (95%CI 0.2–3.4); *p* < 0.035; *I*^2^ = 0.0%]. On the contrary, neither BMI (*p* = 0.78), nor the ASA classification (*p* = 0.90), nor the total amount of pre-SA fluid administered (*p* = 0.11), nor the total amount of pre-SA SBP (*p* = 0.73) had an impact on the incidence of hypotension after SA. Data regarding preoperative hypertension of enrolled patients (reported by two studies [29, 34]), home use of beta-blockers (reported by two studies [33]), and intraoperative blood loss (reported by one study [32]) were unsuitable for meta-regression analysis (Supplemental Table 7 in the Online Supplement Content).

## Discussion

The main findings of the present study in patients undergoing lower limb surgery may be summarized as follows: (1) In RCTs reporting comparable intraoperative effectiveness, in elderly patients undergoing surgical repair of hip fracture, a dose of 6.5 mg of SA for elderly patients was effective and associated with a lower incidence of hypotension, as compared to a dose of 10.5 mg. (2) Hyperbaric SA was associated with a higher incidence of hypotension.

SA technique has been extensively investigated in the past in different clinical settings, with the primary purpose of balancing intraoperative effectiveness (i.e., the minimal effective dose determining a successful sensory and motor block) and systemic side effects. However, overall the comparability of clinical trials considering systemic side effects of SA is rather complicated. Several factors, intrinsic to the technique itself (i.e., baricity and dose and volume of local anesthetic agent), related to the enrolled patients (i.e., age, BMI, pre-existing comorbidities) may influence the final effect, as well as prophylactic/rescue treatments put in action to prevent or reduce undesired hemodynamic systemic effects (i.e., fluid loading) [35, 36].

As the matter of fact, the analysis of the impact of all these variables on hypotensive events is rather complex and the results obtained by the meta-regression should be considered with caution. In fact, the number of the included is relatively small and only the variable “baricity” has been consistently reported.

We focused our meta-analysis on homogenous studies on hip fracture orthopedic surgery with a median duration of about 1 h, including a very old population with a median age > 80 years old. All the included studies reported no statistical difference in the effectiveness of SA and no need for adjunctive general anesthesia for surgery.

Except for the study of Errando et al. [29], all the others adopted SA opiate adjuvants. Twenty years ago, Ben-David et al. demonstrated that a “mini-dose” of bupivacaine (4 mg) with fentanyl (20 mcg) as an adjuvant for hip fracture surgical repair in elderly patients provided effective SA and reduced systemic hemodynamic effects, as compared to 10 mg bupivacaine [30]. The use of a low-dose diluted local anesthetic may limit the spread of spinal block, reducing systemic effects, but may not provide an adequate level of sensory block. Adding intrathecal opiates enhances the analgesia provided by subtherapeutic doses of local anesthetics due to synergistic effects, improving also cardiovascular stability and enhancing early ambulation [36–39]. These advantages should be balanced to the known risks correlated to intrathecal opiate administration (nausea, vomiting and pruritus are quite common; urinary retention occurs in 25–36% of patients; sedation/respiratory depression is rare) [40].

Interestingly, except for SA baricity, none of the potential confounding factors analyzed with the meta-regression influenced the incidence of hypotension after SA, strengthening the concept that low dose SA reduces hypotensive episodes, irrespectively of other patient or SA-related factors. Almost 30 years ago, Carpenter et al. identified two main risk factors for developing hypotension after SA (i.e., sensory anesthesia level and age), reporting an overall incidence of this complication of 25–69% in elderly patients [17]. However, bupivacaine’s mean dose in that study was 9 ± 6 mg, whereas the other anesthetics considered (i.e., lidocaine, tetracaine, and prilocaine) are nowadays rarely or no longer used [17]. Attempted lateralization of subarachnoid anesthesia with the patient positioned laterally may also ameliorate hypotension [41, 42]. This approach has been used in all the included studies, except for the study of Olofsson et al. [31].

Baricity differences between SA solutions may affect the distribution within the subarachnoid space, which may, in turn, affect onset, extent, and duration of the sensory block as well as hemodynamic side effects, being hyperbaric solutions more suitable to reach the higher thoracic dermatomes as opposed to their equivalents [35]. The variability in the cerebrospinal fluid volume in the elderly and a wide range of sensory block heights observed in the studies would also play a role [32, 35]. For instance, Errando et al., despite adopting half the dose of hyperbaric bupivacaine 0.5% infused in the study of Olofsson et al., reported a very different block height [29, 31].

### Clinical implications

Due to intrinsic limitations of the included studies (see also below), the clinical interpretation of our results is rather complex. For sure, “one size does not fit all”, since the number of clinical and physiological variables potentially impacting on the hemodynamic effects of the SA. However, our results show that 6.5 mg of SA anesthetics may be considered a reasonable dose to provide both an effective spinal block and to reduce the incidence of hypotensive events. For sure, one of the main variables is the surgical time, which was of about 1 h, on average, in the studies included in the meta-analysis. Interestingly, in the hip fracture repair setting, the SA dose has been reduced in the last 10 years (i.e., from about 10 mg [41, 42], down to 7.5 mg [22]). This may be considered a consequence of the recent robust evidence associating intraoperative hemodynamic instability and postoperative complications and death [21, 22, 25]. Encouraging the use of the smallest dose of SA needed to perform a safe and effective SA, especially in elderly patients, is, therefore, a key message. The correct management of patients with hip fracture is far from being established, considering also the results of a very recent RCT, which found that SA for hip-fracture surgery in older adults was not superior to general anesthesia with respect to survival and recovery of ambulation at 60 days [43].

### Limitations of the study

Firstly, the overall *I*^2^ (56.9%) suggests moderate heterogeneity in the included studies, with two of them [31] exceeding the 95% confidence interval in the funnel plot analysis. This is probably due to the smaller or greater [31] difference in the hypotensive events compared to the other studies, concerning the overall number of patients enrolled. Overall, since this is not an in-patient meta-analysis, the small number of patients included, the high rate of women, and the monocentric design limit the included studies’ generalizability, potentially biasing the results.

Secondly, the definition of hypotension is known to affect the rate of intraoperative hypotensive events [26]. To minimize this bias, we restricted the inclusion only to those studies adopting specific definitions used in the literature to define intraoperative hypotension in terms of absolute values (i.e., SAP < 90 mmHg [21]) and percentage changes from baseline [21, 26]. However, considering a timeline of 20 years of inclusion, the definitions adopted were not identical for all the studies (i.e., Kahloul et al. [33] defined as hypotension a lower SAP drop, as compared to the other studies) and this should be considered as a key bias. Moreover, also the frequency of the intraoperative blood pressure measurements may also impact this outcome. However, all the studies included adopted definitions of intraoperative hypotension frequently used in the literature [23, 25, 26].

In all the included studies but one [29], lipophilic opiates were added to the local anesthetic. As previously discussed, these drugs are known to affect the spread of the anesthetic in the cerebrospinal fluid, enhancing the effectiveness of SA and reducing side effects [17, 24, 44]. Accordingly, our results should be considered taking in account this bias on the systemic hemodynamic effects of SA. Moreover, in the study of Lilot et al. [34], the type of anesthetic used (ropivacaine) is different as compared to the others (bupivacaine). Previous studies in different settings showed an overall equivalence of same doses of these two anesthetics on central and systemic effects [45, 46]; however, the small amount of comparable studies in this field makes further subanalyses regarding this point unsuitable.

Finally, we adopted a database combination search strategy, including PUBMED®, EMBASE®, and the Cochrane Controlled Clinical trials register, excluding different sources (i.e., Web of Science®). Although this choice should allow a reliable coverage of the published studies for the topic of interest, some RCTs could not be identified, since we did not use crowdsourcing.

## Conclusions

This meta-analysis conducted in elderly patients undergoing surgical repair of hip fracture included six studies administering a mean low dose of 6.5 mg of SA, which was effective in producing intraoperative comfort and motor block and associated with a lower incidence of hypotension than a high mean dose of 10.5 mg. Hyperbaric SA was associated with a higher incidence of hypotension. Data interpretation, however, is limited by the small number of patients included in the included studies, by the inconsistency in the dose of local anesthetic and in the definition of hypotension adopted in the included studies.

## Supplementary Information

**Additional file 1: Supplemental Table 1.** PRISMA checklist. **Supplemental Table 2.** EMBASE search strategy. **Supplemental Table 3.** Extracted data in each study assessed for eligibility. **Supplemental Table 4.** Definition of bradycardia and hypotension in the included studies. **Supplemental Table 5.** Full-text articles excluded, not fitting eligibility criteria. **Supplemental Table 6.** Meta-regression analysis.

## Data Availability

The datasets used and/or analyzed during the current study are available from the corresponding author on reasonable request.
