# Supplementary material for: Spinal anesthesia and hypotensive events in hip fracture surgical repair in elderly patients: a meta-analysis
Source: J Anesth Analg Crit Care. 2022 May 8;2:19. doi: 10.1186/s44158-022-00047-6 (PMC10245414; doi:10.1186/s44158-022-00047-6)
Supplement: Supplementary file 1 — Additional file 1: Supplemental Table 1. PRISMA checklist. Supplemental Table 2. EMBASE search strategy. Supplemental Table 3. Extracted data in each study assessed for eligibility. Supplemental Table 4. Definition of bradycardia and hypotension in the included studies. Supplemental Table 5. Full-text articles excluded, not fitting eligibility criteria. Supplemental Table 6. Meta-regression analysis. [file 44158_2022_47_MOESM1_ESM.docx]

**Spinal anesthesia and hypotensive events in hip fracture surgical repair in elderly patients: a meta-analysis.**

Short title: hypotension following spinal anesthesia.

Antonio Messina, MD, PhD^1,2^; Angelo Milani, MD^1^; Marzia Savi, MD^1^; Filippo Sanfilippo, MD^3^; Katerina Negri, MD^1^; Gianluca Castellani, MD^1^; Gianmaria Cammarota, MD, PhD^3^; Manuela Morenghi^1,2^; Marinella Astuto, MD^4,5^; Maurizio Cecconi, MD, FRCA, FICM^1,2^.

^1^Humanitas Clinical and Research Center – IRCCS, Milano, Italy; ^2^Department of Biomedical Sciences, Humanitas University, Pieve Emanuele, MI, Italy; ^3^Department of Anesthesia and Intensive Care Medicine, Maggiore della Carità University Hospital, Novara, Italy; ^4^Department of Anaesthesia and Intensive Care, A.O.U. "Policlinico-San Marco", Catania, Italy; ^5^School of Anaesthesia and Intensive Care, University Hospital "G. Rodolico", University of Catania, Catania, Italy.

**Corresponding author:**

Antonio Messina, MD, PhD. Department of Anaesthesia and Intensive Care Medicine;

IRCCS Humanitas, Humanitas University; Via Alessandro Manzoni, 56 - 20089 - Rozzano (Milano -Italy).

Tel: +39(0)2 8224 1

email: [antonio.messina@humanitas.it](mailto:antonio.messina@humanitas.it)

**ELECTRONIC SUPPLEMENTARY MATERIALS**

**Table of Contents**

**Supplemental Table 1.** PRISMA checklist.

**Supplemental Table 2.** EMBASE search strategy.

**Supplemental Table 3.** Extracted data in each study assessed for eligibility.

**Supplemental Table 4.** Definition of bradycardia and hypotension in the included studies.

**Supplemental Table 5.** Full-text articles excluded, not fitting eligibility criteria.

**Supplemental Table 6.** Meta-regression analysis.

Supplemental Table 1. PRISMA-DTA checklist for systematic review and meta-analysis

| **Section/topic** | **#** | **Checklist item** | **Reported on page #** |
| --- | --- | --- | --- |
| **TITLE** | | |  |
| Title | 1 | Identify the report as a systematic review, meta-analysis, or both. | 1 |
| **ABSTRACT** | | |  |
| Structured summary | 2 | Provide a structured summary including, as applicable: background; objectives; data sources; study eligibility criteria, participants, and interventions; study appraisal and synthesis methods; results; limitations; conclusions and implications of key findings; systematic review registration number. | 3-5 |
| **INTRODUCTION** | | |  |
| Rationale | 3 | Describe the rationale for the review in the context of what is already known. | 5-6 |
| Objectives | 4 | Provide an explicit statement of questions being addressed with reference to participants, interventions, comparisons, outcomes, and study design (PICOS). | 6 |
| **METHODS** | | |  |
| Protocol and registration | 5 | Indicate if a review protocol exists, if and where it can be accessed (e.g., Web address), and, if available, provide registration information, including registration number. | 7 |
| Eligibility criteria | 6 | Specify study characteristics (e.g., PICOS, length of follow-up), and report characteristics (e.g., years considered, language, publication status) used as criteria for eligibility, giving rationale. | 7-8 |
| Information sources | 7 | Describe all information sources (e.g., databases with dates of coverage, contact with study authors to identify additional studies) in the search and date last searched. | 7-8 |
| Search | 8 | Present full electronic search strategy for at least one database, including any limits used, such that it could be repeated. | 7-8 |
| Study selection | 9 | State the process for selecting studies (i.e., screening, eligibility, included in systematic review, and, if applicable, included in the meta-analysis). | 7-8 |
| Data collection process | 10 | Describe method of data extraction from reports (e.g., piloted forms, independently, in duplicate) and any processes for obtaining and confirming data from investigators. | 7-8 |
| Data items | 11 | List and define all variables for which data were sought (e.g., PICOS, funding sources) and any assumptions and simplifications made. | 8-9 |
| Risk of bias in individual studies | 12 | Describe methods used for assessing risk of bias of individual studies (including specification of whether this was done at the study or outcome level), and how this information is to be used in any data synthesis. | 9 |
| Summary measures | 13 | State the principal summary measures (e.g., risk ratio, difference in means). | 9 |
| Synthesis of results | 14 | Describe the methods of handling data and combining results of studies, if done, including measures of consistency (e.g., I^2^) for each meta-analysis. | 9-10 |

Page 1 of 2

| **Section/topic** | **#** | **Checklist item** | **Reported on page #** |
| --- | --- | --- | --- |
| Risk of bias across studies | 15 | Specify any assessment of risk of bias that may affect the cumulative evidence (e.g., publication bias, selective reporting within studies). | 9-10 |
| Additional analyses | 16 | Describe methods of additional analyses (e.g., sensitivity or subgroup analyses, meta-regression), if done, indicating which were pre-specified. | 9-10 |
| **RESULTS** | | |  |
| Study selection | 17 | Give numbers of studies screened, assessed for eligibility, and included in the review, with reasons for exclusions at each stage, ideally with a flow diagram. | 11-12 |
| Study characteristics | 18 | For each study, present characteristics for which data were extracted (e.g., study size, PICOS, follow-up period) and provide the citations. | 11-12 |
| Risk of bias within studies | 19 | Present data on risk of bias of each study and, if available, any outcome level assessment (see item 12). | 11-12 |
| Results of individual studies | 20 | For all outcomes considered (benefits or harms), present, for each study: (a) simple summary data for each intervention group (b) effect estimates and confidence intervals, ideally with a forest plot. | 11-12 |
| Synthesis of results | 21 | Present results of each meta-analysis done, including confidence intervals and measures of consistency. | 11-12 |
| Risk of bias across studies | 22 | Present results of any assessment of risk of bias across studies (see Item 15). | 11-12 |
| Additional analysis | 23 | Give results of additional analyses, if done (e.g., sensitivity or subgroup analyses, meta-regression [see Item 16]). | 11-12 |
| **DISCUSSION** | | |  |
| Summary of evidence | 24 | Summarize the main findings including the strength of evidence for each main outcome; consider their relevance to key groups (e.g., healthcare providers, users, and policy makers). | 14-15 |
| Limitations | 25 | Discuss limitations at study and outcome level (e.g., risk of bias), and at review-level (e.g., incomplete retrieval of identified research, reporting bias). | 15-16 |
| Conclusions | 26 | Provide a general interpretation of the results in the context of other evidence, and implications for future research. | 17 |
| **FUNDING** | | |  |
| Funding | 27 | Describe sources of funding for the systematic review and other support (e.g., supply of data); role of funders for the systematic review. | 2 |

Supplemental Table 2. EMBASE search strategy

| **Queries** | **Session Results** |
| --- | --- |
| #1. ('hypotension'/exp OR hypotension) AND 'spinal anesthesia’ | 4,282 |
| #2 AND 'Article'/it AND 'human'/de | 2,615 |
| #3 AND 'Article'/it AND 'human'/de AND ([adult]/lim OR [aged]/lim OR [middle aged]/lim OR [very elderly]/lim OR [young adult]/lim) | 2,085 |

Supplemental Table 3. Extracted data in each study assessed for eligibility.

| Study Reference | Names and surnames of authors, year of publication. |
| --- | --- |
| Study design | Modality of recruitment |
| Gender | Percentage of male patients |
| Age | Patient age reported in the study (as mean ± sd or median). |
| Patients characteristics and peri-operatory risk. | Body mass index; height; weight  (as mean ± sd or median). American Society of Anesthesiologists physical status. |
| Patients’ enrollment | Number of enrolled/eligible/dropped out patients in the two study groups |
| Spinal anesthesia characteristics | Type of local anesthetic, dosage, and volume. Site of injection, the needle used, the patient's position, fluid administered during the procedure, adjuvants used with local anesthetics. |
| Hemodynamic data | MAP, SAP, and HR at baseline and 5-10-15 minutes after the procedure. |
| Complications | Hypotension, bradycardia, nausea, and vomiting rates. |

MAP, mean arterial pressure; SAP, systolic arterial pressure; HR, heart rate.

Supplemental Table 4. Bradycardia and Hypotension definition in the studies.

| **Authors** | **Year** | **Bradycardia** | **Hypotension** |
| --- | --- | --- | --- |
| **Errando et al. ^1^** | 2014 | - | SBP < 90 mmHg or < 100 mmHg (if basal > 160 mmHg) |
|  |  |  |  |
| **Olofsson et al. ^2^** | 2011 | - | SBP < 90 mmHg or reduction > 25% of baseline |
|  |  |  |  |
| **Ben David et al. ^3^** | 2000 | - | SBP < 90 mmHg or reduction > 25% of baseline |
|  |  |  |  |
| **Lilot et al. ^4^** | 2013 | HR < 45 bpm | SBP reduction > 30% of baseline |
|  |  |  |  |
|  |  |  |  |
|  |  |  |  |
| **Martyr et al. ^5^** | 2005 | - | SBP < 90 mmHg or reduction > 25% of baseline |
|  |  |  |  |
| **Kahloul et al. ^6^** | 2017 | - | SBP reduction > 20% of baseline |
|  |  |  |  |

HR, heart rate; SBP; systolic blood pressure.

Supplemental Table 5. Full-text articles excluded, not fitting eligibility criteria

| Kallio et al | Br J Anaesth. 2004 |
| --- | --- |
| Liu et al | Anesth Analg. 1997 |
| Kuusniemi et al | Reg Anesth. 1997 |
| Casati A et al | Acta Anaesthesiol Scand. 2000 |
| Cappelleri G et al | Minerva Anestesiol. 2000 |
| Ben-David B et al | Anesth Analg. 2000 |
| Casati A et al | Can J Anaesth. 1997 |
| Marhofer P et al | Reg Anesth Pain Med. 1999 |
| de Visme V et al | Reg Anesth Pain Med. 2000 |
| Wang H et al | J Coll Physicians Surg Pak. 2019 |
| Kuusniemi KS et al | Reg Anesth Pain Med. 2001 |
| Fattorini F et al | Minerva Anestesiol. 2006 |
| Danelli G et al | Can J Anaesth. 2008 |
| Black AS et al | Br J Anaesth. 2011 |
| Mung'ayi V et al | Afr Health Sci. 2015 |
| del-Rio-Vellosillo M et al | Biomed Res Int. 2014 |
| Moosavi Tekye SM et al | Braz J Anesthesiol. 2014 |
| Donati A et al | Minerva Anestesiol. 2007 |
| Tay DH et al | Anaesth Intensive Care. 1992 |
| Fettes PD et al | Br J Anaesth. 2005 Jan |
| Whiteside JB et al | Br J Anaesth. 2003 |
| Mohta M et al | Anaesthesia. 2010 |
| Olsen KH et al | Br J Anaesth. 1990 |
| Wlody D. et al | Am J Obstet Gynecol. 1995 |
| Kafle SK et al | Can J Anaesth. 1994 |
| Filos KS et al | Anesthesiology. 1994 |
| Baraka AS et al | Anesth Analg. 1994 |
| Rahrle G et al | Clin Sci (Lond). 1996 |
| Casati A et al | Reg Anesth. 1996 |
| Veering BT et al | Br J Anaesth. 1996 |
| De Negri P et al | Minerva Anestesiol. 1997 |
| Arndt JO et al | Anesth Analg. 1998 |
| Galimberti G et al | Minerva Anestesiol. 1999 |
| Malinovsky JM et al | Anesthesiology. 1999 |
| Lim HH et al | Anesth Analg. 2000 |
| Malinovsky JM et al | Anesth Analg. 2000 |
| Veering BT et al | Br J Anaesth. 2001 |
| Goel S et al | Eur J Anaesthesiol. 2003 |
| Asehnoune K et al | Anesth Analg. 2005 |
| Nishikawa K et al | J Clin Monit Comput. 2007 |
| Cuvas O et al | Anaesthesia. 2009 |
| Kuusniemi KS et al | Anaesthesia. 1999 |
| Povey HM et al | Acta Anaesthesiol Scand. 1995 |
| Singh J et al | Kathmandu Univ Med J (KUMJ). |
| Gulec D et al | J Int Med Res. 2014 |
| Lee YY et al | Eur J Anaesthesiol. 2005 |
| Iannuzzi E et al | Minerva Anestesiol. 2004 |
| ToptaÅŸ M et al | Turk J Med Sci. 2014 |
| Taivainen T et al | Br J Anaesth. 1990 |
| Imbelloni LE et al | Anaesth Intensive Care. 2009 |
| Nishiyama T et al | J Anesth. 2003 |
| Spasiano A et al | Minerva Anestesiol. 2007 |
| Demirel I et al | Eur Rev Med Pharmacol Sci. 2014 |
| Maurer K et al | Acta Anaesthesiol Scand. 2003 |
| Kelly JD et al | Eur J Anaesthesiol. 2005 |
| Hussien RM et al | Korean J Anesthesiol. 2019 |
| Kiasari AZ et al | Med Arch. 2017 |
| Riesmeier A et al | Anesth Analg. 2009 |
| Cantark M et al | Rev Bras Anestesiol. 2012 |
| Pawlowski J et al | Anesth Analg. 2000 |
| Gaggero et al | Acta Anaesthesiol Scand. 1993 |
| Favarel-Garrigues et al | Anesth Analg. 1996 |
| Davarci I et al | J Int Med Res. 2013 |
| Kuusniemi et al | Anaesthesia 1999 |
| Esmaoglu et al | Acta Anaesthesiol Scand. 1998 |
| Owcuzuk et al | Journal of Regional Anesthesiology 2014 |
| Fathi et al | Anesthesia and Pain medicine 2013 |
| Shin et al | Regional anesthesia and pain medicine 2015 |
| Khezri et al | Acta anesthesiologica Taiwanica 2012 |
| Casati et al | Regional anesthesia and pain medicine 1999 |
| Boztug et al | Journal of Clinical Anesthesia 2006 |
| Horlocker et al | Anesthesia analgesia 1994 |
| Jarvela et al | Anesthesia analgesia 2000 |
| Buggy et al | Anesthesia and Analgesia 1997 |
| Kuusniemi | Regional anesthesia and pain medicine 2000 |
| Buggy et al | Anesthesia and Analgesia 1997 |
| Faust et al | Anesthesia and Analgesia 2003 |
| Olofsson et al | Anesthesiology 2011 |
| Kaya et al | Regional anesthesia and pain medicine 2004 |
| Van der Linden et al | Society of Cardiovascular Anesthesiology 2011 |
| Atef et al | Local and regional anesthesia 2012 |
| Zorko et al | Anesthesia analgesia 2009 |
| Kaya et al | Anesthesia and intensive care 2010 |
| Unal et al | Journal of pakistani medicine 2012 |
| Casati et al | European Journal of anesthesiology 2003 |
| Kamenik et al | Regional anesthesia and pain medicine 2001 |
| Ertuk et al. | Medical Principles and Practice 2010 |

Supplemental Table 6. Reasons for the risk of bias judgements.

|  | Risk of bias judgement |
| --- | --- |
| Errando et al | Randomized, double-blind clinical trial, ITT analysis |
| Ben David et al | Randomized, double-blind clinical trial, ITT analysis |
| Oloffson et al | Randomized, double-blind clinical trial, ITT analysis |
| Martyr et al | Randomized, double-blind clinical trial, ITT analysis |
| Kahloul et al | No predefined analysis strategy is stated. Trial protocol not available |
| Lilot et al | Randomized, single-blinded trial |

Supplemental Table 7. Meta-regression analysis.

| **VARIABLE: Body Mass Index (BMI)**  Meta-regression Number of obs = 4  REML estimate of between-study variance tau2 = 1.141  % residual variation due to heterogeneity I-squared_res = 64.54%  Proportion of between-study variance explained Adj R-squared = -58.98%  With Knapp-Hartung modification  ------------------------------------------------------------------------------  log_ORglob1 \| Coef. Std. Err. t P>\|t\| [95% Conf. Interval]  -------------+----------------------------------------------------------------  bmi \| -.2133441 .6841793 -0.31 0.785 -3.15713 2.730442  _cons \| 2.784802 16.26885 0.17 0.880 -67.2144 72.784  ------------------------------------------------------------------------------  **VARIABLE: American Society of Anesthesiologists (ASA) classification**  Meta-regression Number of obs = 4  REML estimate of between-study variance tau2 = .6883  % residual variation due to heterogeneity I-squared_res = 57.35%  Proportion of between-study variance explained Adj R-squared = -115.74%  With Knapp-Hartung modification  ------------------------------------------------------------------------------  log_ORglob1 \| Coef. Std. Err. t P>\|t\| [95% Conf. Interval]  -------------+----------------------------------------------------------------  asa34m50g1 \| -.142947 1.09089 -0.13 0.908 -4.836667 4.550773  _cons \| -2.516574 .7701346 -3.27 0.082 -5.830196 .7970477  ------------------------------------------------------------------------------  **VARIABLE: Fluid administration before spinal anesthesia**  Meta-regression Number of obs = 5  REML estimate of between-study variance tau2 = 0  % residual variation due to heterogeneity I-squared_res = 0.00%  Proportion of between-study variance explained Adj R-squared = 100.00%  With Knapp-Hartung modification  ------------------------------------------------------------------------------  log_ORglob1 \| Coef. Std. Err. t P>\|t\| [95% Conf. Interval]  -------------+----------------------------------------------------------------  totalfluidml \| -.0157381 .0071232 -2.21 0.114 -.0384073 .0069311  _cons \| 4.487551 3.225109 1.39 0.258 -5.776185 14.75129  ------------------------------------------------------------------------------  **VARIABLE: Systolic pressure before spinal anesthesia**  Meta-regression Number of obs = 4  REML estimate of between-study variance tau2 = .7011  % residual variation due to heterogeneity I-squared_res = 55.30%  Proportion of between-study variance explained Adj R-squared = -196.54%  With Knapp-Hartung modification  ------------------------------------------------------------------------------  log_ORglob1 \| Coef. Std. Err. t P>\|t\| [95% Conf. Interval]  -------------+----------------------------------------------------------------  sapb \| -.0670466 .1674793 -0.40 0.728 -.7876518 .6535585  _cons \| 7.873732 26.01114 0.30 0.791 -104.0432 119.7906  ------------------------------------------------------------------------------  **VARIABLE: Hyperbaric/Hypobaric spinal anesthesia**  Meta-regression Number of obs = 6  REML estimate of between-study variance tau2 = 0  % residual variation due to heterogeneity I-squared_res = 0.00%  Proportion of between-study variance explained Adj R-squared = 100.00%  With Knapp-Hartung modification  ------------------------------------------------------------------------------  log_ORglob1 \| Coef. Std. Err. t P>\|t\| [95% Conf. Interval]  -------------+----------------------------------------------------------------  iperbarico01 \| -1.855053 .591602 -3.14 0.035 -3.497604 -.2125026  _cons \| -1.590105 .3201928 -4.97 0.008 -2.479103 -.7011073  ------------------------------------------------------------------------------ |
| --- |

**Bibliography**

1. Errando CL, Soriano-Bru JL, Peiró CM, Ubeda J. Single shot spinal anaesthesia with hypobaric bupivacaine for hip fracture repair surgery in the elderly. Randomized, double blinded comparison of 3.75 mg vs. 7.5 mg. Rev Esp Anestesiol Reanim. Dec 2014;61(10):541-8.

2. Olofsson C, Nygårds EB, Bjersten AB, Hessling A. Low-dose bupivacaine with sufentanil prevents hypotension after spinal anesthesia for hip repair in elderly patients. Acta Anaesthesiol Scand. Nov 2004;48(10):1240-4.

3. Ben-David B, Frankel R, Arzumonov T, Marchevsky Y, Volpin G. Minidose bupivacaine-fentanyl spinal anesthesia for surgical repair of hip fracture in the aged. Anesthesiology. Jan 2000;92(1):6-10.

4. Lilot M, Meuret P, Bouvet L, et al. Hypobaric spinal anesthesia with ropivacaine plus sufentanil for traumatic femoral neck surgery in the elderly: a dose-response study. Anesth Analg. Jul 2013;117(1):259-64.

5. Martyr JW, Stannard KJ, Gillespie G. Spinal-induced hypotension in elderly patients with hip fracture. A comparison of glucose-free bupivacaine with glucose-free bupivacaine and fentanyl. Anaesth Intensive Care. Feb 2005;33(1):64-8.

6. Kahloul M, Nakhli MS, Chouchene A, Chebbi N, Mhamdi S, Naija W. Comparison of two doses of hypobaric bupivacaine in unilateral spinal anesthesia for hip fracture surgery: 5 mg versus 7.5 mg. Pan Afr Med J. 2017;28:108.
